# Low major histocompatibility complex class II DQA diversity in the Giant Panda (*Ailuropoda melanoleuca*)

**DOI:** 10.1186/1471-2156-8-29

**Published:** 2007-06-07

**Authors:** Liang Zhu, Xiang-Dong Ruan, Yun-Fa Ge, Qiu-Hong Wan, Sheng-Guo Fang

**Affiliations:** 1 College of Life Sciences, Zhejiang University, Hangzhou 310058, PR China; 2 State Conservation Center for Gene Resources of Endangered Wildlife and the Key Laboratory of Conservation Genetics and Reproductive Biology for Endangered Wild Animals, Ministry of Education, Hangzhou 310058, PR China

## Abstract

**Background:**

The giant panda (*Ailuropoda melanoleuca*) is one of the most endangered animals due to habitat fragmentation and loss. Although the captive breeding program for this species is now nearly two decades old, researches on the genetic background of such captive populations, especially on adaptive molecular polymorphism of major histocompatibility complex (MHC), are still limited. In this study, we characterized adaptive variation of the giant panda's MHC *DQA *gene by PCR amplification of its antigen-recognizing region (i.e. the exon 2) and subsequent single-strand conformational polymorphism (SSCP) and sequence analyses.

**Results:**

The results revealed a low level of *DQA *exon 2 diversity in this rare animal, presenting 6 alleles from 61 giant panda individuals. The observed polymorphism was restricted to 9 amino acid substitutions, all of which occurred at and adjacent to positions forming the functionally important antigen-binding sites. All the samples were in Hardy-Weinberg proportions. A significantly higher rate of non-synonymous than synonymous substitutions at the antigen-binding sites indicated positive selection for diversity in the locus.

**Conclusion:**

The *DQA *allelic diversity of giant pandas was low relative to other vertebrates. Nonetheless, the pandas exhibited more alleles in *DQA *than those in *DRB*, suggesting the alpha chain genes would play a leading role when coping with certain pathogens and thus should be included in conservation genetic investigation. The microsatellite and MHC loci might predict long-term persistence potential and short-term survival ability, respectively. Consequently, it is recommended to utilize multiple suites of microsatellite markers and multiple MHC loci to detect overall genetic variation in order to design unbiased conservation strategies.

## Background

Genes of the major histocompatability complex (MHC) are known to be involved intimately in the central control of the immune response, influencing host response to infectious disease challenge. These genes are highly polymorphic in vertebrates [1]. This genetic variation alters the peptide-binding site of the encoded proteins, enabling them to bind a variety of foreign peptides [2]. Many studies support the general hypothesis that allelic diversity at MHC genes is maintained by parasite-mediated balancing selection [3-7]. It has been suggested that species with low MHC polymorphism may be particularly vulnerable to infectious diseases [8,9].

**Figure 1:**
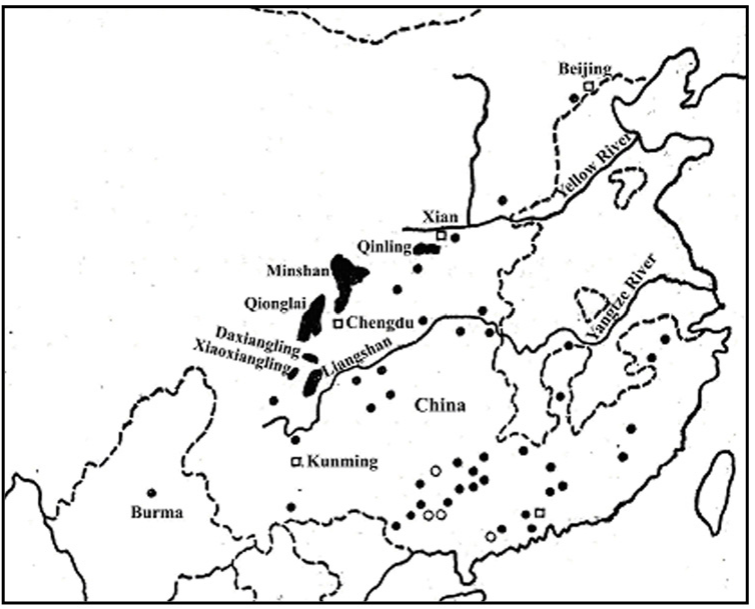
**Current and historical distribution of the giantpanda [11]**. Black areas, present distribution; White circle indicates fossil records in the Early Pleistocene and solid circle shows fossil records in the Mid and Late Pleistocene.

The giant panda (*Ailuropoda melanoleuca*) once had a wide distribution in southwest China, including Hunan, Hubei, Sichuan, Shaanxi and Gansu provinces in the 16–19th centuries. However, habitat destruction and fragmentation have extirpated it from most of its original range [10] and the population of giant panda has decreased sharply. In the 1980s the global population of giant pandas was estimated to be about 1000 [10]. Now giant pandas are restricted to the isolated Qinling, Minshan, Qionglai, Daxiangling, Xiaoxiangling and Liangshan mountains (Figure 1). The historical separation between the Qinling and other populations has yielded a new Qinling subspecies from the nominate Sichuan subspecies [12,13]. A captive breeding program was initiated in 1980's. Now two biggest captive populations are bred in the Ya'an-Wolong and Chengdu breeding bases in Sichuan Province, containing 57 and 86 pandas, respectively [14]. The population size of wild giant pandas of Qinling subspecies was approximately 200 [10], having no captive populations but raising few rescued individuals in Louguantai base.

Pathological researches of captive giant pandas demonstrated that 44% of giant pandas had infected with pneumonia and tick-born disease and the mortality rate of ascariasis had attained to 66.67% [15,16]. This showed that the giant pandas in captivity were particularly susceptible to infectious disease and parasites. Although different neutral DNA marker systems such as mitochondrial DNA [17], minisatellites [12] and microsatellites [18] were used to examine genetic background of giant pandas and revealed that the observed population reduction had a negative impact on genetic variation in the giant panda, neutral molecular markers can not reflect adaptive molecular polymorphism of MHC and thus fail to tell changes in fitness traits [19-21].

The MHC is divided into three classes of genes; one of these, the class II genes, encodes glycoproteins on the surface of cells [5]. Within this gene region, two subregions, DR and DQ, exhibit high levels of polymorphism [22]. Despite a wealth of references documenting allelic polymorphism in carnivore *DQA *genes [23-28], relatively little is known about their counterparts in the bears, especially in the giant panda.

Diversity of MHC *DRB *gene, the most polymorphic locus, has been studied for the giant panda in our laboratory [29]. As a result, in this study, we used the same techniques to investigate genetic variation of another polymorphic *DQA *locus in the giant panda using single-strand conformational polymorphism (SSCP) and sequence analyses. This study provided an insight into the level of giant panda MHC polymorphism and gave some possible implications for captive management of giant pandas.

## Results

**Figure 2:**
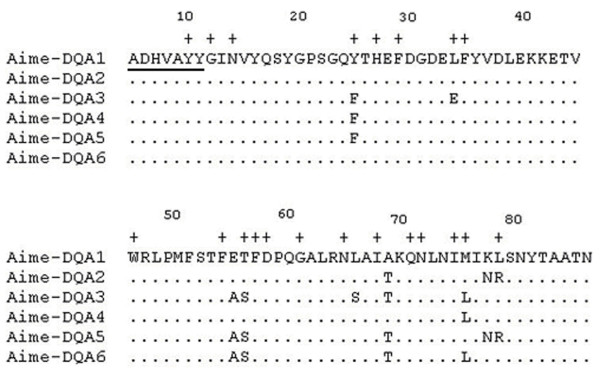
**Comparison of exon 2 amino acid sequences of giant panda *DQA *alleles**. Underlined indicates the upstream primer binding sites (the downstream primer was located on the intron 2 and thus excluded from amino acid sequences). A dot represents identity with the top sequence and a cross indicates putative sites involved in peptide binding as proposed for the human DQα molecules [29].

Sequence variation of *DQA *exon 2 was examined by SSCP, revealing 6 different alleles *Aime-DQA1 *~ *Aime-DQA6 *(Figure 2). All sequences have been deposited in GenBank (Accession number: EF554075-EF4080). The Ya'an-Wolong and Chengdu populations of Sichuan subspecies presented 4 and 5 alleles, respectively, and shared 4 ones with each other (Table 2). The Louguantai population of Qinling subspecies showed 6 alleles, involving all of alleles from Sichuan subspecies (Table 2). The 6 Louguantai alleles showed relatively even frequencies, while alleles *Aime-DQA1 *~ *Aime-DQA5 *were unevenly distributed in both Sichuan populations (Table 2). Table 2 indicated lower observed (*H*_*O*_) than expected (*H*_*E*_) heterozygosities in the studied populations but revealed no significant deviations from Hardy-Weinberg equilibrium in any groups.

**Table 2:** The allelic frequencies, and the observed (*H*_*O*_) and expected (*H*_*E*_) heterozygosities for the three populations of giant pandas.

Subspecies	Location	*Aime*- *DQA*1	*Aime*- *DQA*2	*Aime*- *DQA*3	*Aime*- *DQA*4	*Aime*- *DQA*5	*Aime*- *DQA*6	*H*_*O*_	*H*_*E*_	*P*
Sichuan	Ya'an-Wolong	0.400	0.325	0.000	0.175	0.100	0.000	0.650	0.712	0.242
	Chengdu	0.115	0.173	0.385	0.250	0.077	0.000	0.692	0.755	0.093
Qinling	Louguantai	0.167	0.233	0.133	0.167	0.133	0.167	0.800	0.855	0.822

The polymorphism at class II loci occurs predominately in the exon 2, which encodes a majority of the peptide region [30]. The entire exon 2 translates into a sequence length of 87 amino acids with 21 possible binding sites for foreign peptide presentation [31]. Here the exon 2 region produce 76 amino acids with 20 ABS (Figure 2) if excluding the upstream primer binding sites. Alignments of nucleotide and amino acid sequences showed that the polymorphism was restricted to 11 nucleotide substitutions, together causing 9 amino acid substitutions (Figure 2). All the amino acid variation was found at ABS and adjacent to them (Figure 2). Of the 20 ABS, 5 (25%) were variable over the 6 alleles, whereas only 4 (7.1%) of the remaining 56 positions (those not thought to interact with ABS) were polymorphic. The giant panda *DQA *alleles obtained shared 95.7 to 99.1% nucleotide identity in the 234 bp exon 2 sequences (excluding the primer binding sequences), corresponding to 89.7 to 97.4% amino acid identity. The relative frequency of nonsynonymous substitutions (*d*_*N*_) was significantly higher than that of synonymous ones (*d*_*S*_) in the antigen-binding site for all three populations (Table 3), consistent with the proposed maintenance of high variation by diversifying selection.

**Table 3:** Synonymous and non-synonymous substitutions in the 6 *DQA *alleles of the giant panda. Distances were calculated separately for ABS and non-ABS and the distribution of gene frequency was taken into account. Standard errors were computed using 1000 bootstrap replicates. N is the number of codons in each category and *P *is the probability that *d*_*N *_and *d*_*S *_are different.

Population	Positions	N	*d*_*N*_	*d*_*S*_	*d*_*N*_/*d*_*S*_	*P*
Ya'an-Wolong	ABS	21	0.022 ± 0.013	0.000 ± 0.000	∞	0.047
	Non-ABS	70	0.014 ± 0.007	0.011 ± 0.011	1.273	0.441
	All	91	0.016 ± 0.006	0.010 ± 0.010	1.600	0.259
Chengdu	ABS	21	0.055 ± 0.030	0.000 ± 0.000	∞	0.033
	Non-ABS	70	0.018 ± 0.008	0.009 ± 0.009	2.000	0.169
	All	91	0.027 ± 0.010	0.007 ± 0.007	3.857	0.037
Louguantai	ABS	21	0.044 ± 0.021	0.000 ± 0.000	∞	0.022
	Non-ABS	70	0.017 ± 0.007	0.011 ± 0.011	1.545	0.292
	All	91	0.024 ± 0.008	0.009 ± 0.009	2.667	0.063
All	ABS	21	0.047 ± 0.023	0.000 ± 0.000	∞	0.020
	Non-ABS	70	0.016 ± 0.007	0.010 ± 0.010	1.600	0.214
	All	91	0.024 ± 0.008	0.008 ± 0.008	3.000	0.046

## Discussion

In the human DQα chain (*DQA *gene encoded), residues in the positions 7, 30, 65, 72, 75 and 76 are crucial for protein to form functional conformation and bind a foreign peptide [31]. The amino acid sequences of the giant panda showed identical residues or similar hydrophobic ones to those of human at these positions. None of the nucleotide sequences showed deletions, insertions, or stop codons. Moreover, in the functionally important antigen recognition and binding sites, the polymorphism over all alleles revealed a significantly higher rate of non-synonymous than synonymous substitutions (Table 3; *P *< 0.05), providing evidence for positive selection pressure on these gene loci. Consequently, all of these implied a functional role for these molecules in pathogen-specific immune responses.

The giant panda *DQA *allelic diversity was low compared with that of other vertebrates have been investigated. For instance, the number of alleles in ovin and horse were 24 and 18, respectively, and their numbers of variable amino acids were 37% and 46%, respectively [32,33]. Differently, the giant panda only had 6 *DQA *alleles and 13.6% of variable amino acid positions, indicating that giant pandas had a limited capacity of recognizing diverse pathogens. Some studies showed that polymorphism was more extensive in class II beta chain genes than in the alpha chain genes [34,35], implying that the *DRB *loci should have more alleles than *DQA*. However, our data seems to challenge this prediction. Although the Ya'an-Wolong population had more *DRB *alleles than *DQA *ones, both Chengdu and Louguantai populations had more *DQA *alleles and *DRB *ones (Table 4), suggesting that the alpha chain genes would play a leading role when coping with certain pathogens and thus should be included in genetic investigation when intending to design management strategies.

**Table 4:** Parameters of genetic diversity for the three populations based on different markers.

	Heterozygosity (*Ho/He*)			Average number of alleles per locus		
	Microsatellite	*DRB *[29]	*DQA*	Microsatellite	*DRB *[29]	*DQA*

Ya'an-Wolong	0.57/0.62 [36]	0.64/0.74	0.65/0.71	5.5 [36]	4	3
Chengdu	null/0.64 [37]	0.48/0.60	0.69/0.76	4.5 [37]	4	5
Louguantai	0.57/null [18]	0.73/0.81	0.80/0.86	3.3 [18]	5	6

Microsatellites and MHC are neutral molecular markers and functional genes, respectively, but they both possess high variability and bi-parental genetic information, thus gradually becoming powerful tools in the examination of genetic diversity and population structure. The microsatellite heterozygosity showed that the three populations had similar level of genetic diversity while the heterozygosities of MHC loci indicated that the Louguantai population kept the most abundant genetic variation (Table 4). Regarding the Ya'an-Wolong and Chengdu populations, the *DRB *and *DQA *gave inconsistent results: the former had higher *DRB *heterozygosity but the latter exhibited higher *DQA *heterozygosity (Table 4). From an allelic perspective, comparisons among the number of alleles of different markers also revealed discordant results: average number of alleles for microsatellites was Ya'an-Wolong > Chengdu > Louguantai but that for *DRB *and *DQA *was Louguantai > Chengdu > Ya'an-Wolong (Table 4). The microsatellite-based and *DRB*-based *Fst *values disclosed that significant genetic differentiation existed between Ya'an-Wolong/Chengdu and Louguantai (Table 5), in good agreement with their gene sources, i.e. the Ya'an-Wolong and Chengdu populations were from the nominate Sichuan subspecies while the Louguantai population was from the new Qinling subspecies. On the contrary, the *DQA*-based *Fst *estimates revealed significant intra Sichuan subspecies rather than inter subspecies genetic divergence (Table 5).

**Table 5:** Pairwise *Fst *indices and their significances (in parentheses; NS = non significant) for the three populations revealed by microsatellites, *DRB *and *DQA*.

Pairwise comparison	Microsatellite [18] ^a^	*DRB *[29]	*DQA*
Ya'an-Wolong ~ Chengdu	0.07 (NS)	0.04 (NS)	0.13 (P < 0.05)
Ya'an-Wolong ~ Louguantai	0.18 (P < 0.05)	0.13 (P < 0.05)	0.04 (NS)
Chengdu ~ Louguantai	0.18 (P < 0.05)	0.18 (P < 0.05)	0.04 (NS)

^a^Gene sources of the giant pandas in the Ya'an-Wolong, Chengdu and Louguantai bases were mainly from the Qionglai, Minshan and Qinling mountain ranges. As a result, we utilized the *Fst *indices in the study of Lü *et al*. [18] (0.07 for Minshan ~ Qionglai, 0.18 for Minshan ~ Qinling and 0.18 for Qionglai ~ Qinling).

The inconsistence in heterozygosity, allelic diversity and fixation index among microsatellites, *DRB *and *DQA *should be attributed to the differences in the driving mechanism of polymorphism for different markers and in the identity of individuals sampled in respective studies. The polymorphism of microsatellite loci results from DNA slippage during replication [38] whereas that of MHC genes is pathogen-driven. The neutral variation caused by replication slippage could be accumulated with the evolution of the species, thus predicting long-term evolutionary potential in the face of environmental change. The pathogen-driven MHC polymorphism is dynamic due to continual competition among pathogen variants and the host-pathogen co-evolution, thus being an indicator of ability to cope with short-term pathogen challenges. Scientific conservation plans should consider not only long-term persistence potential but also short-term survival ability. As a result, conservation geneticists should combine lots of microsatellite markers with multiple MHC loci to examine genetic diversity and population structure in order to obtain an overall result and give unbiased management advice. Despite available genetic data from microsatellite, *DRB *and *DQA*, it is a pity that these markers were conducted on different giant panda groups, making it infeasible to design conservation plans from the above-mentioned results at the current stage.

## Conclusion

The *DQA *allelic diversity of giant pandas was low compared with that of other vertebrates have been investigated. Nonetheless, the giant pandas exhibited more alleles in *DQA *than those in *DRB*, suggesting the alpha chain genes would play a critical role when coping with certain pathogens and thus should be included in genetic investigation when intending to design conservation strategies. The microsatellites would accumulate neutral variation whereas the MHC loci could maintain high level of variability during the competition among pathogen variants and the co-evolution between host and pathogen. These two kinds of genetic markers might predict long-term persistence potential and short-term survival ability, respectively. As a result, it is recommended that conservation geneticists should combine the microsatellite markers with multiple MHC loci to examine genetic diversity and population structure in order to obtain an overall result and give unbiased management advice.

## Methods

### Sampling

A total of 61 giant pandas were sampled in this study (Table 1). The samples were obtained from the Ya'an-Wolong Breeding Center (n = 20) and Chengdu Breeding Research Base (n = 26) of Sichuan subspecies and from the Louguantai Saving Center of Rare Wild Animals (n = 15) of Qinling subspecies. Whole blood was collected in routine medical examination and stored at -20°C. Skin samples were collected from dead individuals over last decade and stored at -20°C until use. Faecal samples were collected within 24 hours post-defecation and dried at 65°C overnight. Each dried faecal samples was kept individually in paper bags with silica gel. Genomic DNA was isolated from the blood and skin samples by standard methods [39]. Genomic DNA was extracted from faeces as described by Wan *et al. *[29].

**Table 1:** A list of samples analyzed. A shows the number of adults sampled and B indicated the number of pandas involved in captive breeding program.

Subspecies	Location	Collection year	Sample size	Sample type
Sichuan	Ya'an-Wolong	2004	20 (A = 8; B = 7)	Faeces
	Chengdu	2002 & 2004	14 (A = 10; B = 8)	Blood
		2004	12 (A = 9; B = 7)	Faeces
Qinling	Louguantai	1990–2001	13 (all wild-born)	Skin
		2004	2 (A = 1; B = 1)	Faeces

### PCR amplification

Primers were designed to amplify the second exon of the giant panda *DQA *gene, which presumably encodes for the antigen-binding domain of the cell surface molecule [30]. Primers were designed based on *DQA *consensus regions derived from GenBank sequences (accession number: AY375882 - AY375895, AF343733 - AF343736, U47857 and AJ630363). Primers *DQA-*up (5'-GCT GAC CAT GTT GCT TAC TAT) and *DQA*-down (5'-AAG AGG CAG AGC ATT GGA CA) amplify 275 base pairs. The *DQA *up and down primers hybridize at nucleotide positions 13 to 33 of the exon 2 and 7 to 26 of the intron 2, respectively.

The 20 μL reactions contained 10–100 ng of genomic DNA, 10 × ExTaq buffer (Mg^2+ ^plus) (Takara, Shanghai), 0.2 mM of each dNTP, 0.4 μM of forward and reverse primers, 1 unit of ExTaq (Takarak Shanghai) and 200 μg/mL of bovine serum albumin (Takara, Shanghai) for genomic DNA from faeces. The cycle conditions consisted of an initial denaturation at 95°C for 5 minutes followed by 35 rounds of denaturetion at 95°C for 45 seconds, annealing at 56°C for 45 seconds and extension at 72°C for 45 seconds, with a final extension at 72°C for 10 minutes using a PTC-200 Peltier thermal cycler (M.J. Research Inc. Watertown, MA).Purified PCR product (5 μL) was added to 5 μL 2 × gel loading dye buffer containing 95% formamide, 20 μM DETA, 0.05% bromophenol blue, and 0.05% xylene cyanol and run in 0.5 × TBE (4.5 M TRIS, 4.5 M boric acid, 1 mM EDTA) for 15–18 hours at 8°C. The SSCP bands were visualized by silver staining. The PCR-SSCP analysis was conducted three times for each DNA sample and the results were compared to each other.

Cloning and sequence analysisAt least three examples of each allele were cut separately from the gel with a scalpel knife. The DNA was extracted from these gel strips using acrylamaide gel DNA purification kit (Tianwei, Shanghai), and 2 μL was used in PCR re-amplification. PCR products were separated on a 1.5% agarose gel, recovered using the Agarose Gel Extraction Kit (Takara, Shanghai) and then ligated into pUC18 vector. The inserts of positive clones were verified by SSCP and clones with SSCP patterns identical to the genomic band profile were chosen for sequencing. Nine clones for each allele (from three individuals) were sequenced in both directions, using an ABI 3730 sequencer (Applied Biosystems).

### Data analysis

All nucleotide and amino acid sequences were aligned using ClustalW. MEGA2.1 version was used to estimate the number of synonymous nucleotide substitutions per synonymous site (*d*_*S*_) and the number of nonsynonymous nucleotide substitutions per nonsynonymous site (*d*_*N*_) using the Nei and Gojobori method with a Jukes-Cantor correction [40]. These calculations were performed independently for the nucleotides within and outside the antigen-binding-sites (ABS) [41]). The ABS and non-ABS of these sequences were assigned after Paliakasis *et al*. [30] in accordance with human HLA *DQA *molecule. Expected heterozygosity was calculated after Nei [42] with the small sample size correction and deviation from the Hardy-Weinberg equilibrium was tested using Markov chain permutation test of 100 000 steps in GENEPOP version 3.4 [43]. ARLEQUIN version 2.0 [44] was utilized to calculate *Fst *indices and the significance was tested using 1000 permutations.

## Authors' contributions

LZ and YFG performed the experiments and LZ drafted the first version of the manuscript. XDR collected the samples and drafted the second version of the manuscript. QHW and SGF provided supervision and revised the manuscript. All authors read and approved the final manuscript.
